# Evaluation of postoperative patient satisfaction after covering the nasal dorsum with upper lateral cartilage: “upper lateral closing”^☆^

**DOI:** 10.1016/j.bjorl.2017.10.011

**Published:** 2017-11-16

**Authors:** Can Alper Çağıcı

**Affiliations:** Baskent University Adana Seyhan Hospital, ENT Department, Seyhan, Turkey

**Keywords:** Rhinoplasty, Lateral nasal cartilage, Nasal dorsum, Middle vault, Covering, Rinoplastia, Cartilagem nasal lateral, Dorso nasal, Abóbada média, Cobertura

## Abstract

**Introduction:**

Following nasal hump removal during septorhinoplasty, the middle vault should be reconstructed to avoid functional and esthetic problems. Middle vault reconstruction, however, may result in widening of the middle vault and may need a camouflage graft to cover dorsal irregularities.

**Objective:**

To present the results of reconstructing the middle vault with a technique that covers the nasal dorsum with upper lateral cartilage, from the viewpoint of patient satisfaction.

**Methods:**

Retrospective study of patients who underwent septorhinoplasty that included nasal dorsum closure with upper lateral cartilage from December 1, 2014 to January 31, 2016. Those with postoperative follow-up of less than 3 months were excluded. The final study group included 39 patients. The same surgeon performed all septorhinoplasties. The dorsum was closed using an “upper lateral closing” technique that approximated upper lateral cartilages to each other over the septum. Postoperative patient satisfaction was determined using a visual analog scale and the rhinoplasty outcomes evaluation questionnaire. The questionnaire evaluates patient esthetic and functional satisfaction with the operated nose. High scores indicate improved esthetic results.

**Results:**

No dorsal irregularities were seen at postoperative follow-up evaluation of the patients. For esthetic nasal appearance, the median visual analogue scale scores was 86%, and the mean for the questionnaire was 77.03%.

**Conclusion:**

The natural dome-shaped anatomy of the nasal dorsum was achieved by approximating the upper lateral cartilages to each other. Closing the dorsum with this technique also covers any dorsal irregularities and results in a smooth dorsum. Patients expressed satisfaction with the esthetic and functional aspects of the smooth, attractive nasal dorsum.

## Resumo

**Introdução:**

Após a remoção da giba nasal durante a rinosseptoplastia, a abóbada deve ser reconstruída para evitar problemas funcionais e estéticos. A reconstrução da abóboda entretanto, pode resultar em alargamento dorsal e pode necessitar de um enxerto para camuflar irregularidades dorsais.

**Objetivo:**

Avaliar a satisfação dos pacientes com os resultados da reconstrução da abóboda com uma técnica que utiliza a cartilagem lateral superior para recobrir o dorso nasal.

**Método:**

Estudo retrospectivo de pacientes submetidos a rinosseptoplastia, que incluiu fechamento do dorso nasal com cartilagem lateral superior, realizado de 1° de dezembro de 2014 a 31 de janeiro de 2016. Foram excluídos aqueles com acompanhamento pós-operatório de menos de 3 meses. O grupo final do estudo incluiu 39 pacientes. O mesmo cirurgião realizou todas as rinosseptoplastias. O dorso foi fechado com uma técnica de “fechamento lateral superior” que aproxima as cartilagens laterais superiores de cada lado sobre o septo. A satisfação pós-operatória dos pacientes foi determinada através de uma escala visual analógica e o questionário *Rhinoplasty Outcomes Evaluation*. O questionário avalia a satisfação estética e funcional do paciente com o nariz. Escores altos indicam percepção de melhoria estética.

**Resultados:**

Não foram observadas irregularidades dorsais na avaliação pós-operatória de seguimento dos pacientes. Em relação à aparência nasal estética, o escore médio da escala visual analógica foi 86% e o escore médio do questionário foi 77,03%.

**Conclusões:**

A anatomia natural em forma de domo do dorso nasal foi conseguida através da aproximação das cartilagens laterais superiores entre si. O fechamento do dorso com essa técnica também abrange todas as irregularidades dorsais e resulta em um dorso liso. Os pacientes expressaram satisfação com os aspectos estéticos e funcionais do dorso nasal liso e atraente.

## Introduction

Nasal hump removal is one of the most common procedures during septorhinoplasty. Following hump removal, the middle vault should be reconstructed, especially in patients with a large hump. If this step is not completed, the upper lateral cartilages migrate inferolaterally, possibly resulting in functional and esthetic problems.^1^ Various techniques have been used to address this problem during reconstruction of the middle vault, including primary closure or closing the defect with a spreader graft or spreader flap.

The spreader graft, one of the most popular techniques for closing the middle vault, involves placing a rectangular piece of cartilage between the upper lateral cartilage and the septum.^1^, ^2^, ^3^, ^4^ It spans the point at which the upper lateral cartilages and septum meet. It is quite effective for correcting high septal deviations. This method, however, requires additional cartilage^4^, ^5^ because there may not be an adequate cartilage in the septum, especially in revision cases.^4^ Also, it is technically difficult when using a closed approach.^2^, ^4^, ^5^ In addition, it widens the middle vault,^6^, ^7^ and so would not be preferred in patients with a wide dorsum.

The spreader flap, the most widely accepted middle vault reconstruction technique, entails suturing the medial end of the upper lateral to the septum by folding it in on itself.^2^, ^8^, ^9^, ^10^, ^11^ The most important advantage of this flap is that is does not require additional cartilage tissue. The medial end of the upper lateral cartilage, which is preserved during hump removal, is usually enough cartilage for this technique. The middle vault, however, does not reach the desired width using a spreader graft. To overcome this drawback, various suture techniques were developed for creating spreader flaps.^2^, ^5^, ^10^, ^12^, ^13^

The middle vault is the middle portion of the dorsal esthetic line, which is one of the most important parameters for an esthetically attractive nose. The middle vault is composed of the fused upper lateral cartilage and septum.^3^ Although it is a T-shaped structure at the point of union, the middle vault is dome-shaped in the coronal plane,^9^, ^14^ supported inferiorly by the septum.^9^ A dome-shaped dorsum may not be achieved precisely by either the spreader flap or graft. When using a spreader flap, three cartilages – two upper lateral cartilage flaps and the septum – are brought side by side. This union results in an unnatural plateau of the nasal dorsum. This appearance is more pronounced when using a spreader graft because in spreader grafts five different cartilage segments – two upper lateral cartilages, two spreader grafts, and the septum – are brought side by side.

An alternative technique is thus needed to obtain a natural appearance of the dome-shaped nasal dorsum, which could be obtained by a reconstructive technique that would restore the dorsum to its natural anatomical configuration. One method, which we have now used for 3 years, is to approximate the upper lateral cartilages to each other by suturing them over the septum. This technique restores the natural dome-shaped anatomy of the dorsum without widening it. Patient satisfaction has been high when the ideal natural dorsal profile is achieved, as it is with this technique, which is why we evaluated our postoperative results in regard to the patients’ satisfaction. The aim of this study was to present our algorithm for closing the middle vault with upper lateral cartilage and review our results based on patient satisfaction.

## Methods

We retrospectively evaluated the medical records of patients who underwent septorhinoplasty during which upper lateral cartilage was used for dorsal closure after removing the nasal hump. The same surgeon, who had used this technique since December 1, 2014, performed all septorhinoplasties. We included all patients who underwent nasal hump removal (for esthetic reasons in all cases) and closing of the middle vault. Previous nasal surgery or trauma was not an exclusion criterion. We routinely asked the patients to return for evaluation at 3 and 6 months and 1 year postoperatively, at which time they were asked to complete satisfaction forms.

During the follow-up visits, the patients were asked to mark the Visual Analog Scale (VAS) for the cosmetic appearance (answers ranging from “completely unhappy” to “very happy”) and to fill in the Rhinoplasty Outcomes Evaluation (ROE)^15^ questionnaire. ROE evaluates patients’ esthetic and functional satisfaction about the nose with six questions. All questions are scored from 0 to 4. The total scores may thus be 0–24. These raw scores are divided by 24 and multiplied by 100. Assessments are thus expressed as percentages. High scores mean improved esthetic results.

We retrospectively evaluated the patient's files. We included all patients who filled in the forms at the postoperative follow-up visits. We re-invited patients who did not come for a follow-up visit.

This study was approved by the Baskent University Institutional Review Board (Project n° KA 16/78). We obtained approval from the patients to publish their photographs.

### Surgical technique

We preoperatively evaluated all patients for nasal obstruction. In patients with hypertrophy of the inferior concha, we applied radiofrequency to the inferior concha and lateralized it. We endoscopically excised the lateral lamella of the middle concha bullosa if it was large enough to obstruct the nasal passage. We corrected the septum deviation with septoplasty. We always left an intact and strong “L strut.”

We used an open or closed approach for rhinoplasties. An inverted-V transcolumellar incision was made during open rhinoplasty. Transfixation, infra-cartilaginous, and inter-cartilaginous incisions were used for the closed approach.

The following steps were the same for both open and closed approaches. We elevated the dorsum in the supra-perichondrial and sub-periosteal plane. We bilaterally elevated the mucoperichondrium and mucoperiosteum of the nasal septum. We separated the upper lateral cartilage from the septum by scissors, elevated 1–2 mm of mucoperichondrium under the medial end of the upper lateral cartilages, and excised the cartilage hump with an # 15 blade. We did not excise any cartilage from the upper lateral cartilage at that point. We then removed the bony hump and narrowed the nasal roof via lateral and transverse osteotomies. The cephalic portion of the lower lateral cartilage was then resected and a columellar strut or hemidomal and/or interdomal tip sutures were placed according to need.

### Covering the dorsum with upper lateral cartilage

**Figure 1:**
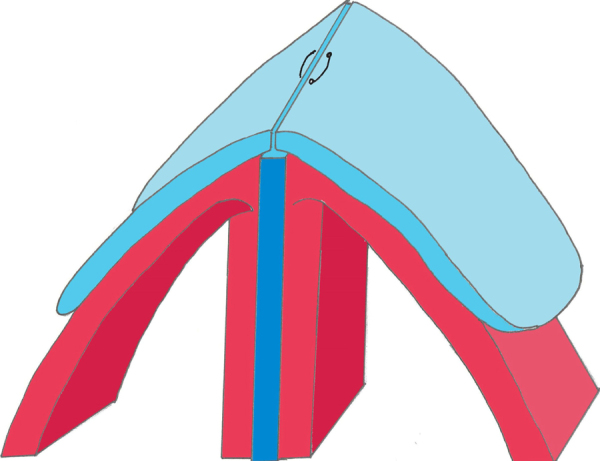
Schema for approximation of the upper lateral cartilages (light blue) over the septum (dark blue). The mucopericondrium is shown in red.

Hereafter, the technique of covering the dorsum with upper lateral cartilage is called “upper lateral closing.” For middle vault reconstruction, we approximated the upper lateral cartilage over the septum with two or three 3/0 polyglactin 910 sutures (Fig. 1). We did not pass the sutures through the septum during this approximation. The dome-shaped normal anatomy of the dorsum was now established. We also tried to ensure that the approximation of the upper lateral cartilages was not too loose or too stretched. We excised the excess parts of the upper lateral cartilage when it was loose. The dorsal height after approximating the upper lateral cartilages over the septum should be evaluated to prevent an iatrogenic hump. We usually removed approximately 1 mm more of the cartilaginous nasal hump than was required.

### Patients with large hump

For large humps, we performed bilateral paramedian wedge resections of the nasal bone. This action helped us approximation of the widely separated upper lateral cartilages with each other.

### Patients with high septum deviation

We corrected high septal deviation using a septal enhancement graft placed more inferiorly than the usual spreader graft. We then proceeded with the “upper lateral closing” over the corrected septum.

### Statistical analysis

Statistical analysis was performed using the statistical package SPSS (Version 17.0; SPSS Inc., Chicago, IL, USA). If continuous variables were normal, they were described as the mean ± standard deviation (*p* > 0.05 with the Kolmogorov–Smirnov test or the Shapira–Wilk test; *n* < 30). If the continuous variables were not normal, they were recorded as the median.

## Results

We have been using the upper lateral closing technique since December 1, 2014 and have applied it in 121 patients (until April 30, 2016). Up to that date, 31 patients had not reached their postoperative 3rd month and so were not included in study. In all, 90 patients completed 3rd postoperative month, but 51 of them did not come to any of the scheduled postoperative follow-up sessions (longer than 3 months). None of these patients were included in study. If the patient came to more than one of the proposed follow-up visits, the results of the latest visit were used. Only 39 patients were finally included in the study.

The upper lateral closing technique was used in all patients (mean ± SD: age 27.84 ± 8.77 years; 29 women; follow-up 7.76 ± 3.99 months). Open approach was used in 36 patients (92.3%). We used septal enhancement grafts, which were placed more inferiorly than the usual spreader grafts, to correct the high septal deviation in nine patients (23%). We did not use the spreader grafts in any patient.

The results of each patient's tests at the latest follow-up visit were collected, and the mean ROE and the median VAS scores for esthetic appearance were calculated. The median VAS score for esthetic appearances of the nose was 86% (scale 0–100%). The mean ROE score was 77.03% ± 17.01%.

**Figure 2:**
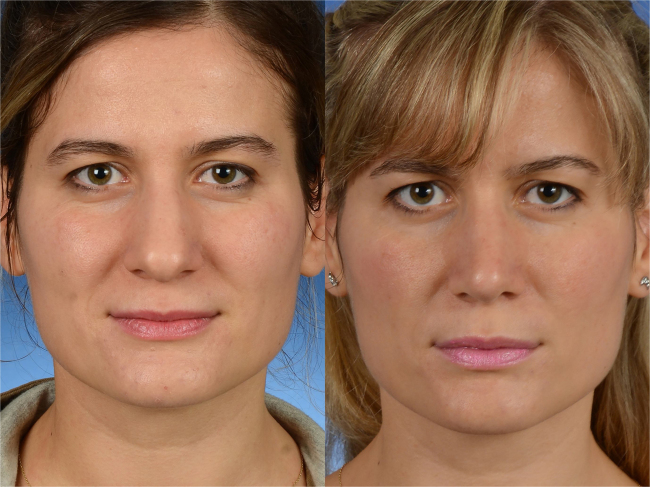
Corrected dorsal esthetic line with normal middle vault wideness is seen preoperative (left) and postoperative (right) frontal views. Her Visual Analog Scale (VAS) score for esthetic appearance of the nose was 86%, and her ROE score was 83%.

**Figure 3:**
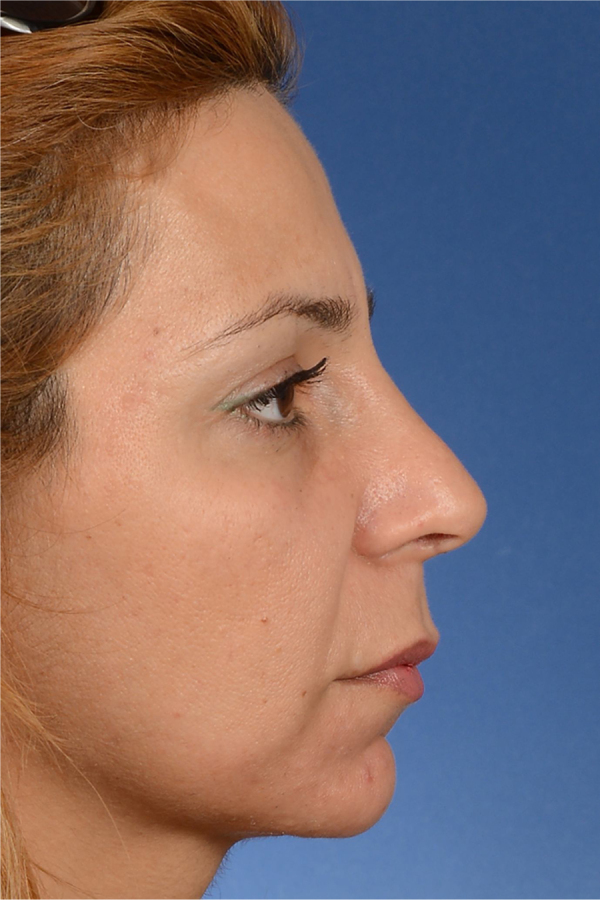
Iatrogenic cartilaginous hump as a result of inadequate adjustment of the septal height. Her VAS score for esthetic appearance of the nose was 86%, and her ROE score was 58%.

**Figure 4:**
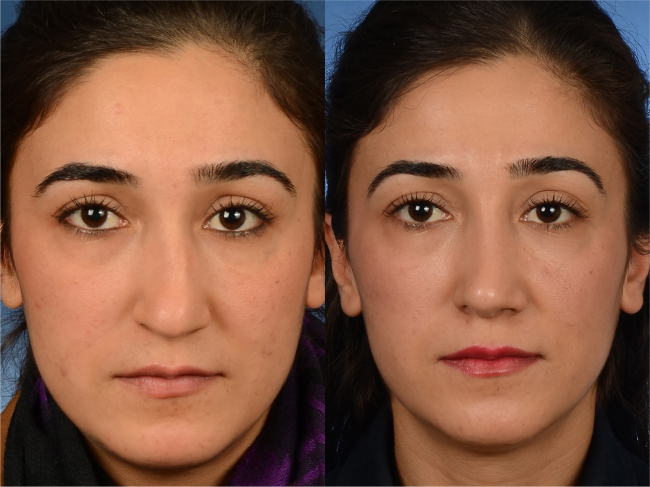
Partially corrected high dorsal deviation. Preoperative (left) and postoperative (right) frontal view of the patient are shown. Her VAS score for esthetic appearance of the nose was 77%, and her ROE score was 63%.

We obtained a dome-shaped smooth dorsum in all patients (Fig. 2). We detected no irregularities on the cartilage dorsum. There were few complications. Four patients had residual bony hump due to inadequate removal. Two patients had an iatrogenic cartilaginous hump because of inadequate adjustment of the septal height (Fig. 3). These patients underwent revision surgery. Another revision was made in a patient because of uncorrected high dorsal deviation caudally (Fig. 4). One patient had an inverted V deformity because of overly- resected upper lateral cartilage.

## Discussion

Nasal hump removal is one of the most commonly used steps in septorhinoplasty. After its removal, the upper lateral cartilages are detached from the septum, causing the upper lateral cartilages to recoil posteroinferiorly.^7^ This situation results in disturbance of the dorsal esthetic line, which if not reconstructed can result in middle vault collapse and an inverted V deformity.^16^ Spreader grafts and flaps are the most widely used techniques for reconstructing the middle vault.^1^, ^2^, ^3^, ^4^, ^8^, ^9^, ^10^ However, they (especially spreader grafts) provide a plateau appearance to the dorsum, which changes the dorsum so it no longer has its natural dome shape, and the brow-tip esthetic line becomes more artificial. Spreader grafts require additional cartilage tissue, whereas spreader flaps do not.^4^, ^5^ Occasionally, camouflage grafts may be needed to cover dorsal irregularities.^3^, ^4^ The “upper lateral closing” technique does not widen the middle vault, does not require additional cartilage tissue, and does maintain the dome-shaped natural anatomy of the dorsum. “Upper lateral closing” provides good esthetic and functional results, which in this study were supported by the ROE and VAS scores. There was no need for camouflage grafts to hide dorsal irregularities because they were covered by upper lateral cartilage.

Patient satisfaction with the results of our rhinoplasties was high. The postoperative mean ROE was 77.03% and the median VAS score for esthetic appearance of the nose was 86%. A dome-shaped appearance of the middle vault was achieved in all patients. Palpation revealed no dorsal irregularities in any of patients. Complications included an inverted V deformity in one of the patients because of overly- resected upper lateral cartilage. Two patients had an iatrogenic cartilaginous hump because of inadequate adjustment of septal height. Four patients had a slight bony hump because of inadequate removal of the original hump. One patient returned with an uncorrected C-shaped high septal deviation that was due to inadequate correction of a high septal deviation caudally.

To date, the most preferred technique for high septal deviation has been the spreader graft. We used an enhancement graft, however, which was placed more inferiorly than the spreader graft but parallel to it in nine patients. First, we adjusted the septal height. Then, we corrected the high septal deviation (if present) using a septal enhancement graft. Later, we closed the dorsum with the “upper lateral closing” technique. Previously, Sciuto and Bernardeschi^17^ had positioned the spreader grafts more inferiorly to decrease widening of the middle vault, and they closed the septum by approximating the upper lateral cartilages over the spreaders in a manner similar to ours. One of our patients returned with an uncorrected C-shaped high septal deviation for which we had previously used a septal enhancement graft (Fig. 4). The problem in this patient was the lack of septal enhancement near the anterior septal angle. We corrected the deviation using a septal extension that was placed more caudally during the revision surgery.

After nasal hump removal in patients with a large hump, the upper lateral cartilages, similar to nasal bones, stay apart from each other. It is not easy to reapproximate them. Roostaeian et al.^7^ had advised “tension spanning suture,” which advances the upper laterals beyond the septum, with the cartilage returning to its original position in patient with big humps. They advised primary reconstruction of the middle vault without using “tension spanning suture” if the patient has a minimal hump with strong cartilage. Although they advised primary closure only in patients with a small hump, we successfully closed the dorsum with “upper lateral closing” in patients with a large hump as well. In those patients, we undertook a paramedian wedge resection from the nasal bone, which helps the extremely separated upper lateral come together. These two techniques, “tension spanning suture” plus paramedian wedge resection may be combined in patient with large humps, although we are very happy about the results achieved using our technique.

Some irregularities on the dorsum are seen after hump removal or dorsum reconstruction.^3^, ^6^, ^7^ There may also be some irregularities during the healing period even on a successfully reconstructed dorsum. Camouflage grafts and flaps are used to cover these irregularities.^3^, ^4^ With the “upper lateral closing” technique, the upper lateral cartilage covers these irregularities, so there is no need for camouflage grafts. To date, however, palpation has revealed no cartilage irregularities in these patients. Hence, in addition to restoring the original anatomy with high esthetic outcomes, supported by high VAS and ROE scores, “upper lateral closing” provides smooth dorsum.

“Upper lateral closing” also adds some height to the septum because upper lateral cartilages are approximated over the septum. This should be taken into consideration when adjusting the dorsal height. If not, inadequate adjustment may result in an iatrogenic nasal hump (Fig. 3). We saw this complication in two of our patients and corrected it during revision surgery.

Covering the dorsum with upper lateral cartilage is not a new technique. Sciuto and Bernardeschi^17^ had described “upper lateral cartilage suspension” to overcome internal valve compromise. In their technique, the upper lateral cartilage is suspended over a dorsal spreader graft. This method is a combination of a spreader graft and “upper lateral closing”. They advised that this technique be applied only in patients with internal nasal valve collapse. Fayman and Potgieter^18^ had also brought the upper lateral cartilages together in a “vest-over” fashion to overcome dorsal irregularities and middle vault narrowing, but it is not end-to-end approximation. Dayan and Greene^19^ had used a similar closing technique that was similar to ours. They used this technique only in patients with thin skin to obtain a smooth, natural nasal dorsum. Roostaeian et al.^7^ had advised nearly the same closing technique for the middle vault in patients with small humps and strong cartilage. We have been successfully using this “upper lateral closing” technique in our patients regardless of skin thickness and hump size.

The most important drawback of the study is that it was retrospective, so it was not possible to use randomization to overcome selection bias. However, we did not select any patients, including those who completed the 3-month postoperative follow-up evaluation. Another limitation is that we do not yet know the long-term results of this technique. We are expecting that the long-term functional and esthetic results of the study will be good because of the favorable short-term results. It would be interesting to compare this technique directly with other available techniques in a prospective study.

## Conclusion

The “upper lateral closing” technique, which reconstructs the natural dome-shaped anatomy of the dorsum, also achieves a smooth dorsum. It is an easy, reproducible technique for closing the middle vault. For more definitive conclusions, we must await the long-term results.

## Funding

This research did not receive any specific grant from funding agencies in the public, commercial, or not-for-profit sectors.

## Conflicts of interest

The author declares no conflicts of interest.
